# Dietary L-leucine supplementation improves ruminal fermentation parameters and epithelium development in fattening Angus beef cattle

**DOI:** 10.1186/s40104-025-01190-0

**Published:** 2025-04-23

**Authors:** Jishan An, Yu Ge, Huitian He, Hao Ge, Jing Li, Zhiqing Li, Lei Liu, Zuo Wang, Xinyi Lan, Weijun Shen, Anwei Cheng, Fachun Wan

**Affiliations:** 1 College of Animal Science and Technology, Hunan Agricultural University, Changsha, Hunan 410128 People’s Republic of China; 2 Yuelushan Laboratory, Changsha, 410128 China; 3 College of Veterinary Medicine, Hunan Agricultural University, Changsha, Hunan 410128 People’s Republic of China; 4 College of Food Science and Technology, Hunan Agricultural University, Changsha, Hunan 410128 People’s Republic of China

**Keywords:** Beef cattle, Leucine, Rumen epithelial morphology, Rumen fermentation, Ruminal microbiome and metabolome

## Abstract

**Background:**

In this study, the effects of L-leucine (Leu) on rumen fermentation parameters, rumen epithelium development, amino acid composition, rumen bacterial communities and rumen metabolites in beef cattle were investigated. Twenty-four fattening Angus females of similar initial weight (575.5 ± 22.1 kg) were randomly assigned to 2 treatments with 4 replicate pens (3 cattle per pen). They were fed either a basal diet or a basal diet supplemented with 6.0 g L-Leu/100 kg BW/d for 120 d.

**Results:**

(1) Leu increased the ruminal concentrations of total volatile fatty acid (VFA) (*P* = 0.017), propionate (*P* = 0.023), isovalerate (*P* = 0.001), and branched-chain volatile fatty acid (BCVFA) (*P* = 0.01) at 4 h post-feeding. It also tended to increase acetate (*P* = 0.083) and decrease the ammonia-N (NH_3_-N) concentration (*P* = 0.055), but it did not affect ruminal pH (*P* > 0.1). Leu also increased microbial crude protein (MCP) (*P* = 0.026) at 4 h post-feeding, but decreased MCP at 8 h post-feeding (*P* = 0.010). (2) Supplementation with L-Leu increased the ruminal concentrations of phenylalanine (*P* = 0.011), lysine (*P* = 0.034), and tyrosine (*P* = 0.033), while decreasing the cystine concentration (*P* = 0.010). (3) Leu increased the thickness of the stratum spinosum and basal (*P* < 0.05), while decreasing the thickness of the stratum granulosum (*P* < 0.05). (4) Leu upregulated the relative mRNA abundance of genes involved in tight junction proteins (*P* < 0.05) and VFA absorption and metabolism (*P* < 0.01) in the rumen epithelium. This upregulation was positively correlated with the concentrations ruminal isovalerate and BCVFA (*P* < 0.01). (5) L-Leu did not affect the diversity and richness of ruminal microbes (*P* > 0.05), but differential bacterial biomarkers (LEfSe, LDA > 2) were either positively or negatively correlated with ruminal MCP, NH_3_-N, and BCVFA concentrations (*P* < 0.001). Additionally, differential bacterial metabolites (OPLS-DA, VIP > 1.5) were primarily enriched in the amino acid metabolism pathway and the cofactors and vitamins metabolism pathway (*P* < 0.05).

**Conclusions:**

Dietary supplementation with L-Leu altered rumen fermentation parameters and patterns, improved rumen epithelial morphology, and enhanced the expression of genes related to VFA absorption and metabolism in the rumen epithelium of beef cattle.

**Supplementary Information:**

The online version contains supplementary material available at 10.1186/s40104-025-01190-0.

## Introduction

In ruminants, rumen fermentation is an effective means of extracting dietary nutrients and is one of the key indicators of changes in rumen function. For functional amino acid nutrition strategies in ruminants, rumen-protected amino acids are used in beef cattle production due to the rumen’s ability to degrade feed crude protein and amino acids [1–3]. However, consistent results have not been obtained, and the role of functional amino acids in rumen development is largely overlooked.

Leucine (Leu), the most abundant of the branched-chain amino acids (BCAAs), has seen only limited progress in rumen function research [4]. Leu is also an essential functional amino acid for beef cattle, primarily derived from rumen microbial crude protein (MCP), rumen undegraded feed protein, and dietary supplements such as L-type crystalline Leu [5, 6]. Results from in vitro rumen fermentation experiments showed that the removal of all BCAAs resulted in suppressed the growth of ruminal microorganisms, decreased fiber degradation, and reduced rumen MCP and volatile fatty acid (VFA) production [7–9], indicating a direct effect of BCAAs on rumen microorganisms and fermentation parameters. This was associated with increased rumen microbial activity of BCAA (Leu) as a nitrogen source. Available studies have confirmed that Leu and isovalerate can be interconverted in the rumen, with isovalerate serving as an essential growth factor for numerous rumen fiber-degrading microorganisms. These microorganisms can alter rumen fermentation patterns, improve VFA concentrations, and enhance MCP synthesis [7, 9–11]. Moreover, the degradation products of leucine, such as α-ketoisocaproate and isovalerate, can influence the absorption and metabolic functions of rumen epithelial cells [8, 10, 11], thereby indirectly regulating rumen fermentation and pH. Generally, high levels of VFAs in rumen fluid can affect rumen microecology and rumen function, while a healthy rumen epithelium can dynamically balance VFA absorption and metabolism [12]. However, limited research has been conducted on the impact of BCAAs and branched-chain volatile fatty acids (BCVFAs) on rumen epithelial cells. It is hypothesized that BCAAs and their metabolites (VFAs) may promote the morphology and development of these cells [13, 14]. Further in vivo animal experiments are necessary to validate this hypothesis. Additionally, increased MCP synthesis in rumen fluid is moved into the lower digestive tract with digesta, which may be an effective way to improve leucine bioavailability for ruminants [2, 15], although this remains to be verified in beef cattle.

To the best of our knowledge, research on Leu has primarily focused on dairy ruminants using post-ruminal and jugular infusion methods, with more attention given to intestinal digestive enzymes and their effects on dairy products [4]. Even fewer studies have reported on the effects of Leu on rumen fermentation function in beef cattle. Based on our previous findings comparing the effects of rumen-protected Leu and L-Leu on in vitro rumen fermentation [7], the improved average daily gain in beef cattle in response to Leu [16], and the findings of others [9, 17], we hypothesized that L-Leu facilitates rumen fermentation and rumen epithelium function in beef cattle. The experiment aimed to study the effects of dietary supplementation with L-Leu on rumen fermentation parameters and amino acid composition, rumen epithelial morphology and gene expression related to VFA absorption and metabolism, and rumen bacterial communities and metabolites in fattening beef cattle.

## Materials and methods

### Animal experimental design

Twenty-four fattening Black Angus females (24 months old, body weight [BW] = 575.5 ± 22.1 kg) were randomly assigned to 2 treatment groups with 4 replicates (pens) per treatment (14 m^2^ per animal). The dietary treatment groups were fed either a basal diet (CON) or a basal diet supplemented with 6.0 g L-Leu/100 kg BW/d (Leu) based on the initial average body weight of each group. The basal total mixed ration (TMR), with a concentrate-to-forage ratio of 70:30, was formulated to meet or exceed the Nutrient Requirements of Beef Cattle [18] (Table 1). The total experimental period lasted 135 d. During the 15-day adaptation period, cattle were gradually transitioned to the high-concentrate diet by replacing 10% of the pre-experimental diet with the new diet each day, based on total dry matter intake. Throughout the 120-day treatment period, each treatment group of animals received its corresponding dietary treatment. All cattle were fed fresh feed daily at 08:00 and 17:00 and had free access to fresh water.

**Table 1:** Composition and nutrient contents of total mixed rations (DM basis)

Item	Content	Composition of concentrate supplement, %	
Ingredient, %		Corn	72.20
Rice straw	30.00	Rice bran	2.00
Concentrate supplement	70.00	Soybean meal	13.30
Total	100	Soybean oil	1.00
Analyzed chemical composition^1^,%		Wheat bran	6.00
Crude protein^2^	12.01	Salt	1.00
Metabolism energy^3^, MJ/kg	11.57	Limestone	1.00
Calcium	0.46	Montmorillonite	0.20
Phosphorus	0.32	CaHPO_4_	0.50
Neutral detergent fiber	30.07	NaHCO_3_	1.00
Acid detergent fiber	14.36	Premix^4^	1.80
Total digestible nutrients^3^	78.90	Total	100
Leucine	0.60		

Performed in duplicate on a composite derived from 4 samples (one sample collected per 30 day)

^1^Chemical composition: metabolism energy and total digestible nutrients were a calculated value, while other values were measured

^2^Calculated as nitrogen × 6.25

^3^Total digestible nutrients and metabolizable energy was calculated according to the Nutrient Requirements of Beef Cattle [18]

^4^Premix contained the following ingredients per kilogram of diet: vitamin A ≥ 120 kIU, vitamin D_3_ ≥ 100 kIU, vitamin E ≥ 300 IU, vitamin K_3_ ≥ 1,250 mg, Mn ≥ 1,500 mg, Zn ≥ 2,000 mg, Cu ≥ 1,500 mg, Fe ≥ 4,000 mg, I ≥ 12 mg, Co ≥ 20 mg, Se ≥ 10 mg

L-Leucine (food grade, ≥ 98% purity) was purchased from Hebei Huayang Biotechnology Co., Ltd. The supplement level of L-Leu used in this study was based on previously published literature [19, 20] and our previous in vitro rumen study [7]. In this study, the precisely weighed L-Leu product was first mixed with a small amount of TMR and provided to each beef cattle in a small basin for consumption, after which the remaining TMR was offered.

### Sample collection

During the last 3 days of the experimental period, 6 cattle were randomly selected from each treatment group for rumen fluid sampling and subsequent slaughter, with each beef cattle serving as the experimental unit. TMR diets were collected at 30 d intervals during the treatment period.

#### Rumen fluid

The rumen fluid was collected from each beef cattle using an esophageal tube at 2 h before the morning feeding, as well as 4 and 8 h post-feeding, over a period of 3 d. The first cup of rumen fluid was discarded, and approximately 100 mL of the second cup was collected as the sample. The pH of the rumen fluid was immediately measured using a pH meter (Seven2Go; Mettler Toledo Technology Co., Ltd., Shanghai, China). The rumen fluid sample was then filtered through 4 layers of cheesecloth, dispensed into eight 5-mL RNA- and DNA-enzyme-free cryopreservation tubes, and stored at −80 °C for rumen fermentation parameters, free amino acids, metabolomics, and metagenomics analysis [21].

#### Rumen epithelial tissue and intestinal contents

On d 121, after 12 h fast, the cattle were slaughtered at a commercial slaughterhouse following standard slaughter procedures [22]. The rumen was immediately separated, and its contents were removed. Subsequently, two segments of epithelial tissue from the ventral sac of the rumen were collected after removing the muscular and serosal layers, then immediately washed in ice-cold phosphate-buffered saline solution until cleared. One ruminal epithelial sample was fixed in 4% paraformaldehyde for histomorphology analysis. Another epithelial sample was cut into pieces, divided into three 5-mL RNA- and DNA- enzyme-free cryopreservation tubes, and stored in liquid nitrogen for RNA extraction and analysis [12, 23].

### Chemical analyses

#### Chemical analyses of TMR diet

The chemical composition of the TMR was analyzed according to the guidelines outlined by the Association of Official Analytical Chemists [24]. Dry matter (DM; 105 °C), crude protein (CP; No. 988.05), and ether extract (EE; No. 922.06) in the TMR diet were analyzed. Total nitrogen was analyzed using the Dumas combustion method (D60; Hanon Technology Development Co., Ltd., Shandong, China), and CP was calculated using a 6.25 nitrogen-to-protein conversion factor. Ether extract content was determined using a Soxhlet apparatus with petroleum ether as the extraction solvent. Neutral detergent fiber (NDF) in TMR diet was analyzed [25], with the inclusion of heat-stable alpha-amylase. Acid detergent fiber (ADF) was analyzed using an Ankom A200i fiber analyzer (Ankom Technology, Macedon, NY, USA). The contents of calcium (No. 977.29) and phosphorus (No. 995.11) were analyzed with the AOAC methods [24].

#### Ruminal fermentation parameters analysis

Ruminal volatile fatty acid (VFA) concentrations were analyzed using a gas chromatograph (GC-8600, Agilent Technologies Inc., USA) equipped with a DB-WAX UI column (30 m × 0.25 mm × 0.25 μm) following the procedures described by Wang et al. [26]. Ruminal ammonia nitrogen (NH_3_-N) concentration was analyzed using a microplate spectrophotometer [Multiskan SkyHigh; Thermo Fisher Scientific (China) Co., Ltd.] based on the method of Broderick et al. [27]. The MCP concentration in rumen fluid was analyzed using the colorimetric method of Makkar et al. [28] on a microplate spectrophotometer [Multiskan SkyHigh; Thermo Fisher Scientific (China) Co., Ltd.].

#### Ruminal free amnio acid

The free amino acids (FAAs) in the rumen liquid were determined using an automatic amino acid analyzer (L-8900, Hitachi Technologies, Inc., Tokyo, Japan). Briefly, the rumen liquid was centrifuged at 12,000 r/min for 15 min at 4 °C, and an aliquot of the supernatant was mixed (1:1) with a 10% trichloroacetic acid solution and vortexed for 1 min. Afterward, the supernatant was collected following centrifugation at 12,000 r/min for 15 min at 4 °C, filtered through a 0.22-μm filter membrane, and transferred into an autosampler vial [7].

#### Metagenomics analysis in rumen fluid samples and data analysis

The quality and quantity of total genomic DNA from rumen fluid were assessed using 1% agarose gel electrophoresis. The extracted DNA samples were fragmented to an average size of approximately 350 bp using a Covaris M220 (Gene Company Limited, China) for paired-end (PE) library construction. A PE library was constructed using the TruSeq DNA Sample Prep Kit following the manufacturer’s instructions (Illumina). PE sequencing was performed using the Illumina HiSeq 4000 platform at Majorbio Bioinformatics Technology Co., Ltd. (Shanghai, China). Adapter sequences were removed from the 3′ and 5′ ends of the paired-end Illumina read using SeqPrep (version 1.1; https://github.com/jstjohn/SeqPrep). Quality control of each dataset was performed using Sickle (version 1.33) to trim low-quality bases (quality scores < 20), and remove short reads (< 50 bp) and “N” records. The filtered reads were de novo assembled for each sample using Megahit (v1.0.6). MetaGene was used to predict open reading frames (ORFs) from the assembled contigs with length > 300 bp. The assembled contigs were then pooled, and non-redundancies contigs were constructed based on the identical contigs using CD-HIT with 95% identity. The information on the abundance of individual genes in different samples was counted and normalized to obtain the gene abundance table. Species composition analysis was based on reads using DIAMOND, compared to the NCBI NR database and combined with RefSeq parsing. Taxonomic profiles were conducted at the domain, phylum, genus, and species levels, with relative abundances calculated. A PCoA based on Bray-Curtis dissimilarity matrices at the species level was also performed [29].

The predicted nonredundant gene sets were compared with the functional annotation databases, the Kyoto Encyclopedia of Genes and Genomes (KEGG) and Carbohydrate-Active enZymes (CAZy). The overall number of non-redundant genes annotated, as well as the number in each sample, was counted [30].

#### Rumen fluid metabolites analysis by metabolomics

Metabolome analysis was conducted using ultra-performance liquid chromatography tandem mass spectrometry (UPLC-MS/MS) [31]. Approximately 100 μL of the rumen fluid samples were transferred into centrifuge tubes (1.5 mL) and mixed with 300 μL of methanol and 10 μL of internal standard (2.8 mg/mL, DL-o-Chlorophenylalanine). Then, the mixture was vortexed for 30 s (Votex-5, Kylin-Bell Lab Instruments Co., Ltd., Haimen, China), kept for 1 h, and centrifuged at 13,000 × *g* and 4 °C for 15 min. In the end, 200 μL of supernatant was transferred into a vial for liquid chromatography-mass spectrometry (LC-MS) analysis. The data were analyzed through the free online platform of Majorbio Cloud Platform (https://cloud.majorbio.com/page/tools).

Orthogonal partial least squares discriminant analysis (OPLS-DA) with minimal supervision was conducted to reduce and classify the collected metabolomics data. The model’s validity was evaluated using model parameters R^2^X, R^2^Y, and Q^2^, which provide information on the interpretability and predictability, respectively, and help avoid the risk of over-fitting. Statistically significant differences among groups were identified with a VIP value greater than 1 and a *P* value less than 0.05. The differential metabolites were further identified and validated through KEGG. Enrichment analysis of the metabolic pathways was performed based on the differential metabolites using the KEGG pathway database.

#### RNA extraction and measured of rumen epithelial tissue

The total RNA from the rumen epithelial tissue across all samples was extracted using a SteadyPure RNA extraction kit (column membrane purification method; AG21024, Accurate Biotechnology (Hunan) Co., Ltd., Changsha, China) according to the manufacturer’s instructions. A spectrophotometer (NanoDrop 2000; Thermo Fisher Scientific, USA) was then used to quantify the RNA concentration, and all samples had an absorption ratio of 260/280 nm between 1.90 and 2.10, which indicates high RNA purity. The integrity of the RNA samples was evaluated using a 1.0% agarose-formaldehyde gel electrophoresis. Afterward, the concentration of each RNA sample was adjusted to 500 ng/μL based on optical density and then stored at −80 °C. A total of 1 μg of high-quality RNA per sample was reverse-transcribed using a Prime Script RT reagent Kit with a gDNA Eraser (AG11707, Accurate Biotechnology (Hunan) Co., Ltd., Changsha, China) according to the manufacturer’s instructions.

The primers for monocarboxylate transporter isoform 1 (*MCT1*), monocarboxylate transporter isoform 4 (*MCT4*), putative anion transporter isoform 1 (*PAT1*), downregulated in adenoma (*DRA*), sodium/proton exchanger isoforms 1, 2, and 3 (*NHE1*, *NHE2*, *NHE3*, respectively), and beta-hydroxybutyrate dehydrogenase isoforms 1 and 2 (*BDH-1*, *BDH-2*), 3-hydroxy-3-methylglutaryl-CoA lyase (*HMGCL*), 3-hydroxy-3-methylglutaryl-CoA synthase isoform 1 and 2 (*HMGCS1*, *HMGCS2*), claudin 1 (*CLDN1*), occludin (*OCLN*), and zonula occluden 1 (*ZO1*), and glyceraldehyde-3-phosphate dehydrogenase (*GAPDH*) were used as described in the published literature (Additional file 1: Table S1). The primer sequences and their corresponding amplicon sizes were cited from Gao et al. [32] and Dieho et al. [33]. The primer sequences were identified and designed using the National Center for Biotechnology Information. All primers were synthesized by Sangon Biotech Co., Ltd. (Shanghai, China). A BioRad CFX-96 real-time PCR system with fluorescence detection of SYBR Green dye was used to perform quantitative real-time PCR (qRT-PCR) of the target genes and GAPDH. The amplification conditions were as follows: 95 °C for 30 s followed by 40 cycles of 5 s at 95 °C and 30 s at 60 °C (AG11701, Accurate Biology, Changsha, China). All measurements were performed in triplicate. The quantification results showed that these primers had no stray peaks or non-specific amplification. The mRNA abundance of *GAPDH* (a housekeeping gene) was used to normalize the relative amount of each studied mRNA, and the 2^−ΔΔCT^ method was used to analyze the data.

#### Measurements of rumen epithelial papilla morphology

Five samples of rumen epithelium from the ventral sac of each cattle were dehydrated, paraffin-embedded, sliced, and stained with hematoxylin and eosin. The thickness of the total epithelia stratum corneum, stratum granulosum, stratum spinosum, and stratum basal was measured using Image-Pro software (Express 6.0, Media Cybernetics, Bethesda, MD, USA) [34].

### Statistical analysis

Data on rumen fermentation parameters, ruminal amino acid composition, rumen papillae morphology, and the relative mRNA abundance of genes in the rumen epithelium were analyzed using a linear mixed model (SPSS 19.0 software, SPSS Inc., Chicago, IL, USA). Data visualization was performed using GraphPad Prism software (version 8.0.2). In the mixed model, the Leu treatment was the fixed effect, and individual cattle were treated as the random term.

In the rumen microbiome analysis, the non-parametric Mann-Whitney test was used to assess alpha-diversity and the relative abundance of the microbiota. Correlations between rumen fermentation parameters and rumen microbiota or epithelial function genes were calculated using Spearman’s correlation test, implemented on Majorbio Cloud Platform (https://cloud.majorbio.com/page/tools). Linear discriminant analysis effect size (LEfSe) analysis was performed using the online LEfSe analysis tool. In the rumen metabolite analysis, the VIP value from multivariable OPLS-DA analysis and the *P* value from the univariable analysis *t*-test were used to screen for significantly differential metabolites (R software, Version 1.6.2). Metabolic pathway and enrichment analyses were then conducted on MetaboAnalyst 3.0 using the differential metabolites. Differences were considered significant when *P* < 0.05, and tendencies noted were 0.05 < *P* < 0.10. The results are presented as mean values with the standard error of the mean (SEM).

## Results

### Rumen fermentation parameters

Table 2 indicates that supplementing with L-Leu increased the ruminal concentrations of total VFA (*P* = 0.017), propionate (*P* = 0.023), iso-valerate (*P* = 0.001), BCVFA (*P* = 0.01), and MCP (*P* = 0.026) at 4 h post-feeding, and tended to increase the acetate concentration (*P* = 0.083) and decrease the NH_3_-N concentration (*P* = 0.055). At 8 h post-feeding, the concentration of MCP were decreased (*P* = 0.01), while the concentrations of total VFA and individual VFAs showed no significant effect (*P* > 0.1). No significant differences were found in ruminal pH at 2 h before morning feeding and at 4 and 8 h post-feeding (*P* > 0.1).

**Table 2:** Effects of L-Leu on rumen fermentation in beef cattle (*n* = 6)

Item	Time, h^1^	Treatment		SEM^2^	*P*-value
		**CON**	**Leu**		
pH	2	7.29	7.31	0.046	0.829
	4	6.44	6.30	0.080	0.379
	8	6.49	6.60	0.064	0.413
VFA, mmol/L					
Total VFA	2	67.49	70.17	3.563	0.726
	4	123.32^a^	148.41^b^	5.626	0.017
	8	135.95	139.95	4.336	0.667
Acetate (A)	2	48.09	49.91	2.351	0.718
	4	90.47	103.97	3.913	0.083
	8	94.02	95.70	3.053	0.797
Propionate (P)	2	10.37	10.30	0.922	0.969
	4	19.06^a^	25.56^b^	1.513	0.023
	8	23.16	23.14	0.965	0.988
Butyrate	2	5.74	6.50	0.337	0.278
	4	10.63	14.74	1.809	0.276
	8	15.15	16.62	1.516	0.649
Valerate	2	0.58	0.62	0.031	0.579
	4	0.78	0.88	0.514	0.380
	8	1.07	1.10	0.077	0.850
Iso-valerate	2	1.35	1.55	0.135	0.477
	4	1.30^a^	2.20^b^	0.148	0.001
	8	1.41	2.04	0.200	0.127
Iso-butyrate	2	1.33	1.27	0.055	0.583
	4	1.05	1.04	0.070	0.956
	8	1.13	1.34	0.090	0.239
BCVFA	2	3.27	3.45	0.200	0.683
	4	3.15^a^	4.13^b^	0.211	0.010
	8	3.61	4.48	0.324	0.190
A:P	2	4.71	5.94	0.751	0.437
	4	5.21	4.09	0.514	0.296
	8	4.09	4.18	0.135	0.775
NH_3_-N, mg/dL	2	31.19	30.48	1.336	0.805
	4	19.31	14.64	1.246	0.055
	8	13.83	14.64	0.808	0.641
MCP, mg/dL	2	16.49	18.71	0.888	0.226
	4	23.77^a^	27.36^b^	0.850	0.026
	8	38.45^a^	31.54^b^	1.475	0.010

*CON* Basal diet group, *Leu* Basal diet supplementation with 6.0 g L-Leu/100 kg BW/d group, *VFA* Volatile fatty acid, *BCVFA* Branched-chain VFA, *A:P* Acetate to propionate ratio, *NH*_*3*_*-N* Ammonia nitrogen, *MCP* Microbial crude protein,

^1^2: 2 h before morning feeding; 4/8: 4/8 h post-feeding

^2^Standard error of the mean

^a,b^Different superscript letters within the same row indicate significant differences (*P* < 0.05)

### Free amino acid concentrations in rumen fluid

Table 3 indicates that supplementing with L-Leu increased the ruminal concentrations of phenylalanine (*P* = 0.011), lysine (*P* = 0.034), and tyrosine (*P* = 0.033), and decreased the concentrations of cystine (*P* = 0.010). It also tended to increase the concentrations of threonine (*P* = 0.058), isoleucine (*P* = 0.064), total essential amino acids (*P* = 0.085), and histidine (*P* = 0.066). No significant differences were found for the concentrations of other amino acids (*P* > 0.1).

**Table 3:** Effects of L-Leu on ruminal free amino acid concentrations at 4 h post-feeding in beef cattle (*n* = 6)

**Item, µg/mL**	**Treatment**		**SEM**^**1**^	***P*-value**
	**CON**	**Leu**		
Methionine	1.02	1.35	0.127	0.222
Threonine	3.40	5.09	0.462	0.058
Valine	3.18	3.53	0.232	0.495
Isoleucine	1.90	2.97	0.297	0.064
Leucine	3.45	3.22	0.468	0.831
Phenylalanine	2.42^a^	3.64^b^	0.279	0.011
Lysine	5.97^a^	10.96^b^	1.266	0.034
Total EAA	21.35	30.79	2.772	0.085
Asparagine	2.85	4.17	0.429	0.129
Cystine	0.92^a^	0.55^b^	0.084	0.010
Serine	2.11	2.61	0.197	0.234
Glutamine	15.46	20.95	2.354	0.275
Glycine	1.49	1.86	0.137	0.190
Alanine	5.11	5.82	0.400	0.414
Tyrosine	2.56^a^	3.60^b^	0.261	0.033
Histidine	0.05	0.17	0.032	0.066
Arginine	0.79	0.67	0.132	0.692
Total NEAA	31.36	40.43	3.790	0.261
Total BCAA	8.53	9.73	0.860	0.530
Total AA	52.71	71.22	6.479	0.167

*CON* Basal diet group, *Leu* Basal diet supplementation with 6.0 g L-Leu/100 kg BW/d group, *BCAA* Branched-chain amino acid, *EAA* Essential amino acid, *NEAA* Nonessential amino acid

^1^Standard error of the mean

^a,b^Different superscript letters within the same row indicate significant differences (*P* < 0.05)

### Rumen epithelium gene expression

**Fig. 1:**
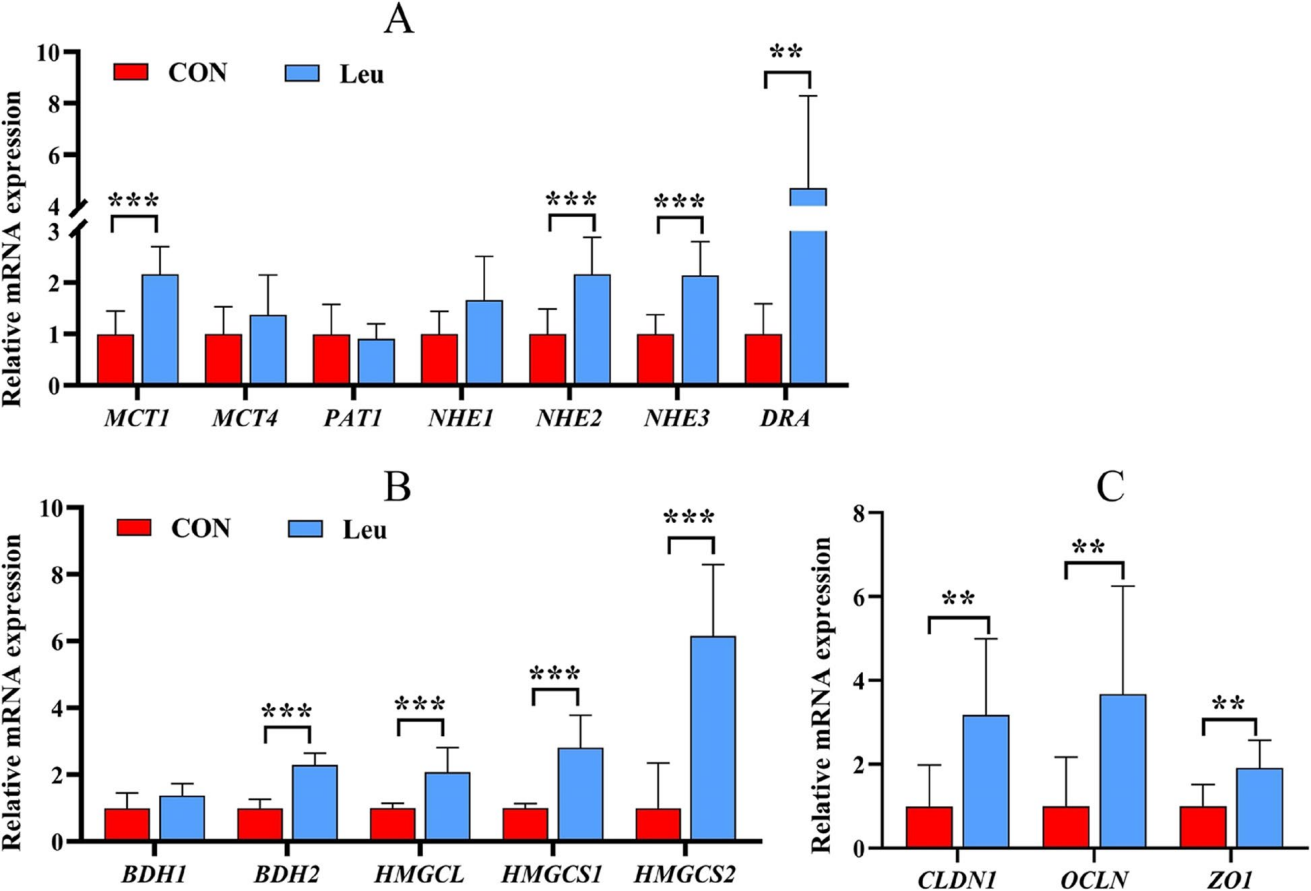
The effects of dietary supplementation L-leucine on messenger RNA (mRNA) expression of genes involved in rumen epithelial (*n* = 6). **A** The relative expression of genes related to VFA absorption in rumen papillae. **B** The relative expression of genes related to VFA metabolism in rumen papillae. **C** The relative expression of genes related to integrity in rumen papillae. Quantitative RT-PCR results were expressed as relative mRNA expression and the data were analyzed by the 2^−ΔΔCT^ method. Significant correlations are shown with ** (*P* < 0.05) and *** (*P* < 0.01). *CON* Basal diet group, *Leu* Basal diet supplementation with 6.0 g L-Leu/100 kg BW/d group, *MCT1/4* Monocarboxylate transporter isoform 1/4, *PAT1* Putative anion transporter isoform 1, *DRA* Downregulated in adenoma, *NHE1/2/3* Sodium/proton exchanger isoform 1/2/3, *BDH 1/2* Beta-hydroxybutyrate dehydrogenase 1/2, *HMGCL* 3-Hydroxy-3-methylglutaryl-CoA lyase, *HMGCS 1/2* 3-Hydroxy-3-methylglutaryl-CoA synthase isoform 1/2

The relative mRNA abundance of genes related to VFA absorption and metabolism in the rumen epithelium and tight junction proteins is shown in Fig. 1 and Additional file 1: Table S2. Supplementing L-Leu increased the relative mRNA abundance of *MCT1* (*P* < 0.01), *NHE2* (*P* < 0.01), *NHE3* (*P* < 0.01), *DRA* (*P* < 0.05), *BDH2* (*P* < 0.01), *HMGCL* (*P* < 0.01), *HMGCS1* (*P* < 0.01), *HMGCS2* (*P* < 0.01), *CLDN1* (*P* < 0.05), *OCLN* (*P* < 0.05), and *ZO1* (*P* < 0.05). No differences were found in the mRNA abundance of *MCT4*, *PAT1*, *NHE1*, and *BDH1* (*P* > 0.1).

### Morphological structure of rumen epithelium

**Fig. 2:**
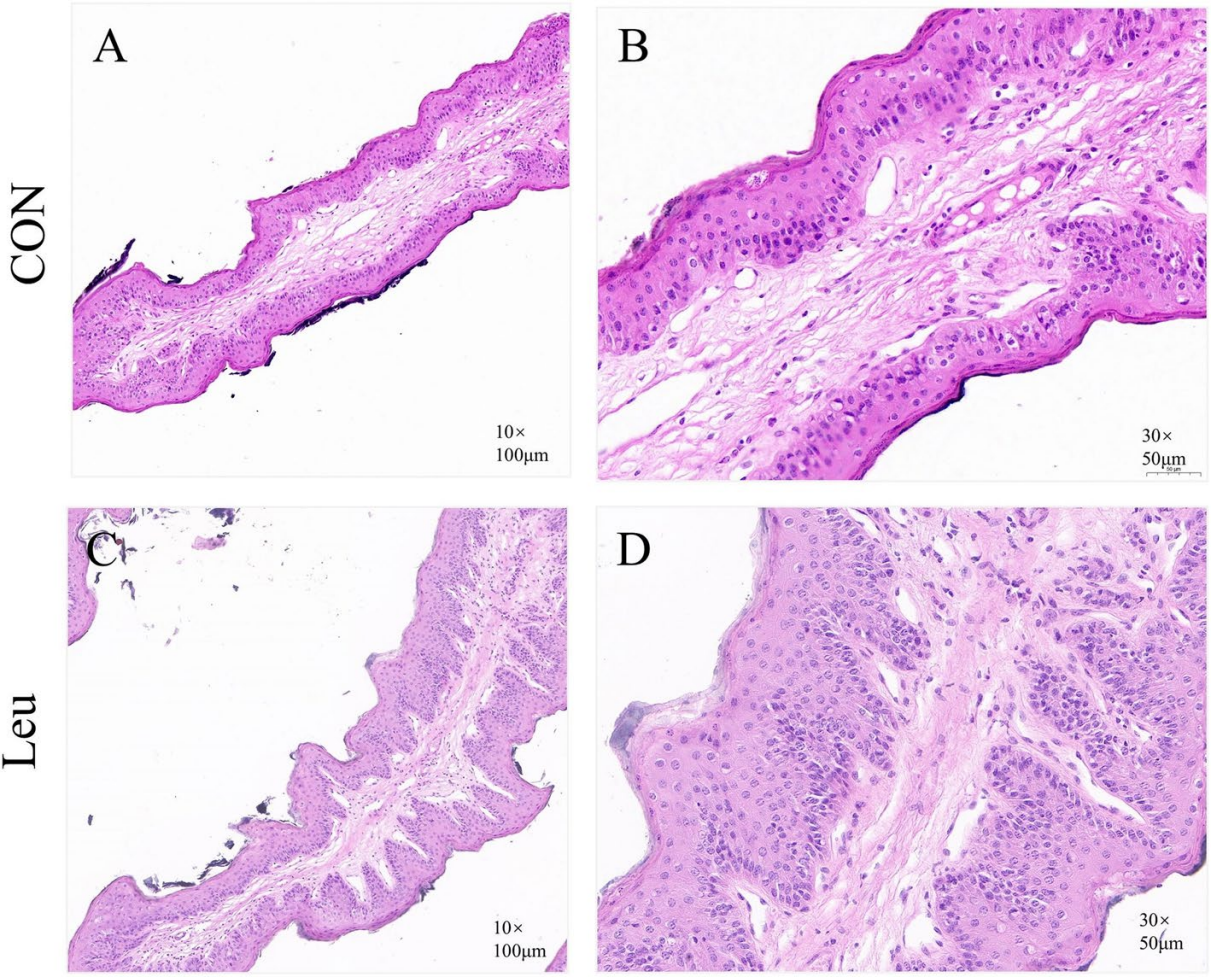
The effect of Leu on ruminal papillae morphology in beef cattle (*n* = 6). Representative rumen epithelial visual graph (**A** and **C**) and micrograph (**B** and **D**) of the beef cattle between CON and Leu. Visual images of the rumen tissues were taken with a camera. Images are obtained through a light micrograph of rumen tissue at a magnification of 10 × and 30 × objective lens. *CON* Basal diet group, *Leu* Basal diet supplementation with 6.0 g L-Leu/100 kg BW/d group

As shown in Table 4 and Fig. 2, dietary supplementation with L-Leu increased the thickness of the stratum spinosum and stratum basal (*P* < 0.05), while decreasing the thickness of the stratum granulosum (*P* < 0.05). No significant differences were found in the thickness of the total epithelia and stratum corneum (*P* > 0.1).

**Table 4:** Effects of L-Leu on ruminal papillae morphology structure in beef cattle (*n* = 6)

Item	Treatment		SEM^1^	*P*-value
	**CON**	**Leu**		
Total epithelia, μm	309.55	353.32	13.79	0.116
Stratum corneum, μm	9.33	9.22	0.240	0.827
Stratum germinativum, μm	14.50^a^	10.82^b^	0.800	0.012
Stratum spinosum and basal, μm	81.46^a^	108.62^b^	6.479	0.028

*CON* Basal diet group, *Leu* Basal diet supplementation with 6.0 g L-Leu/100 kg BW/d group

^1^Standard error of the mean

^a,b^Different superscript letters within the same row indicate significant differences (*P* < 0.05)

### Ruminal microbial community

**Fig. 3:**
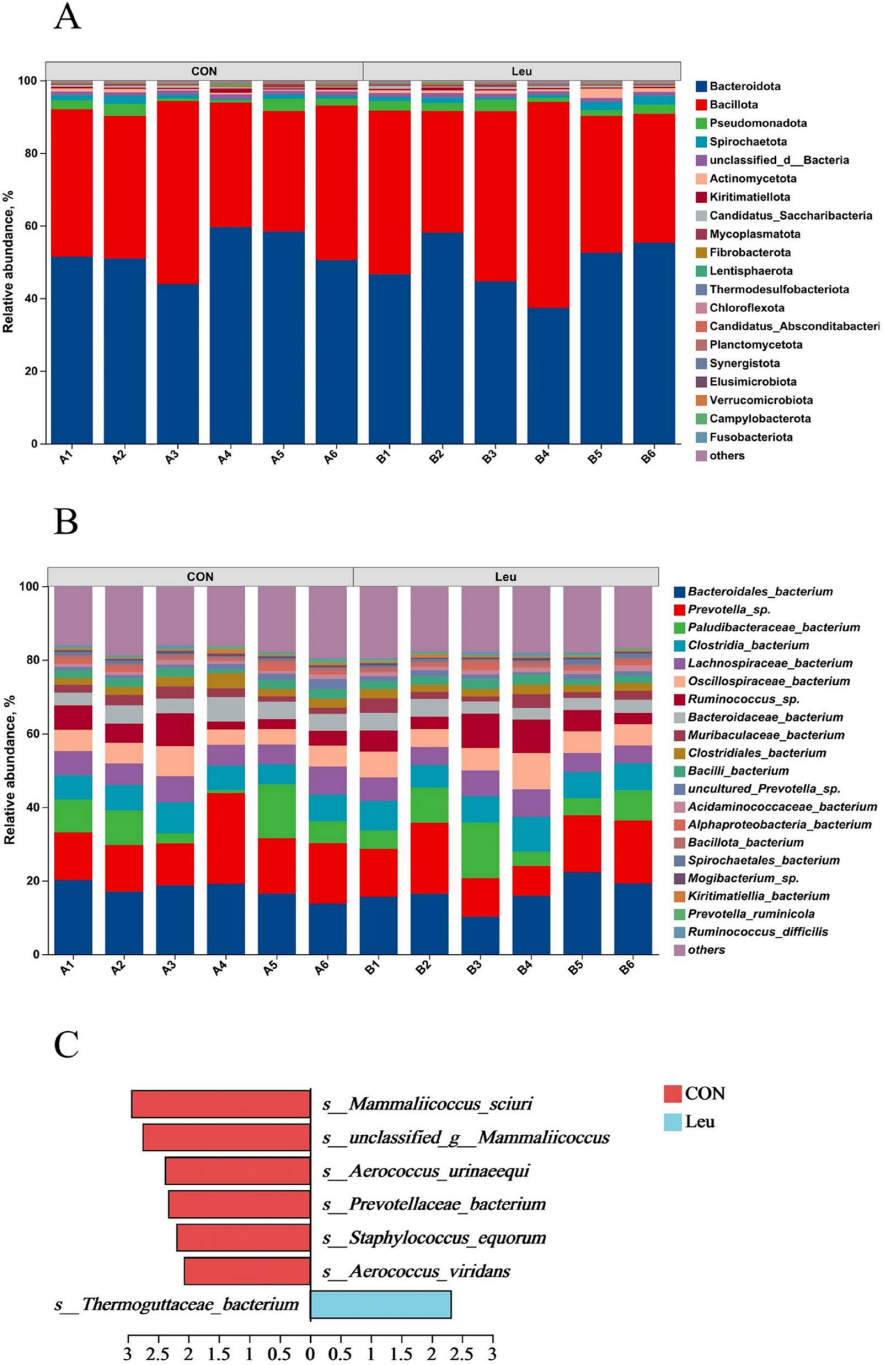
Results of metagenomic sequencing of the rumen bacteria in the CON and Leu (*n* = 6). **A** Differences in bacterial phylum levels. **B** Differences in bacterial species levels. **C** The significantly differential microorganisms based on the linear discriminant analysis effect size (LEfSe) cladogram in metagenomic sequencing. The differences are represented by the color of the group, “CON” means basal diet group (A1–A6); “Leu” means basal diet supplementation with 6.0 g L-Leu/100 kg BW/d group (B1–B6)

Metagenome sequencing generated 45.84 ± 0.44 million raw reads. After quality control and removal of host genes, 45.44 ± 0.44 million clean reads were retained (Additional file 1: Table S3). The Good’s coverage of all samples was greater than 0.99, indicating that the metagenomic sequencing data were sufficient and high accuracy. No significant differences were observed among the alpha-diversity indices, including observed species, Shannon and Chao indices (*P* > 0.05) (Additional file 2: Fig. S1 A and B). Furthermore, supplementing with L-Leu did not affect the community structure, as determined using principal coordinates analysis (PCoA) and non-metric multidimensional scaling (NMDS) (Additional file 2: Fig. S1C and D). At the phylum level, the dominant phyla were Bacteroidota and Bacillota, accounting for 47.26% and 43.9% of total reads, respectively (Fig. 3A; Table 5). Based on Welch’s *t*-test, with a *P*-value threshold of less than 0.05, a total of 8 differential phyla were identified (Additional file 2: Fig. S2 A). Among these differential phyla, 5 had higher abundance in the Leu group, while 3 had lower abundance. At the species level, the most predominant species were *Bacteroidales-bacterium* (17.95%) and *Prevotella_sp.* (13.27%) in the rumen (Fig. 3B; Table 5). Based on Welch’s *t*-test, a total of 15 differential species were identified (Additional file 2: Fig. S2B). LEfSe was used to screen the main specific microorganisms between the two groups, and we found that the relative abundance of *Mammaliicoccus_sciuri*, *unclassified_g_Mammaliicoccus*, *Aerococcus_urinaeequi*, *Prevotellaceae_bacterium*, *Staphylococcus_equorum*, and *Aerococcus_viridans* was greater in the CON group, while *Thermoguttaceae_bacterium* was greater in the Leu group (Fig. 3C; LDA > 2)*.*

**Table 5:** Effects of L-Leu on the relative abundances of the rumen bacterial community at phylum and species in beef cattle (*n* = 6)

Item	Treatment		SEM^1^	*P*-value
	**CON**	**Leu**		
Dominant at phylum (top 5), %				
Bacteroidota	52.50	49.12	1.939	0.410
Bacillota	40.05	42.56	2.124	0.580
Pseudomonadota	2.06	2.19	0.274	0.831
Spirochaetota	1.16	1.48	0.205	0.452
unclassified_d__Bacteria	1.09	1.08	0.030	0.876
Differential (top 3), %				
Planctomycetota	0.05^a^	0.09^b^	0.009	0.031
Candidatus_Melainabacteria	0.006^a^	0.013^b^	0.001	0.013
Myxococcota	0.003^a^	0.002^b^	0.001	0.032
Dominant at species (top 5), %				
*Bacteroidales-bacterium*	17.56	16.63	0.918	0.638
*Prevotella_sp.*	15.55	13.93	1.269	0.548
*Paludibacteraceae_bacterium*	7.09	7.72	1.288	0.820
*Clostridia_bacterium*	6.88	7.53	0.306	0.308
*Lachnospiraceae_bacterium*	6.35	6.00	0.292	0.571
Differential (top 3), %				
*Mammaliicoccus_sciuri*	0.31^a^	0.15^b^	0.036	0.024
*unclassified_g__Mammaliicoccus*	0.20^a^	0.10^b^	0.024	0.026
*Prevotellaceae_bacterium*	0.10^a^	0.06^b^	0.007	0.002

*CON* Basal diet group, *Leu* Basal diet supplementation with 6.0 g L-Leu/100 kg BW/d group

^1^Standard error of the mean

^a,b^Different superscript letters within the same row indicate significant differences (*P* < 0.05)

### Rumen microbiome function

**Fig. 4:**
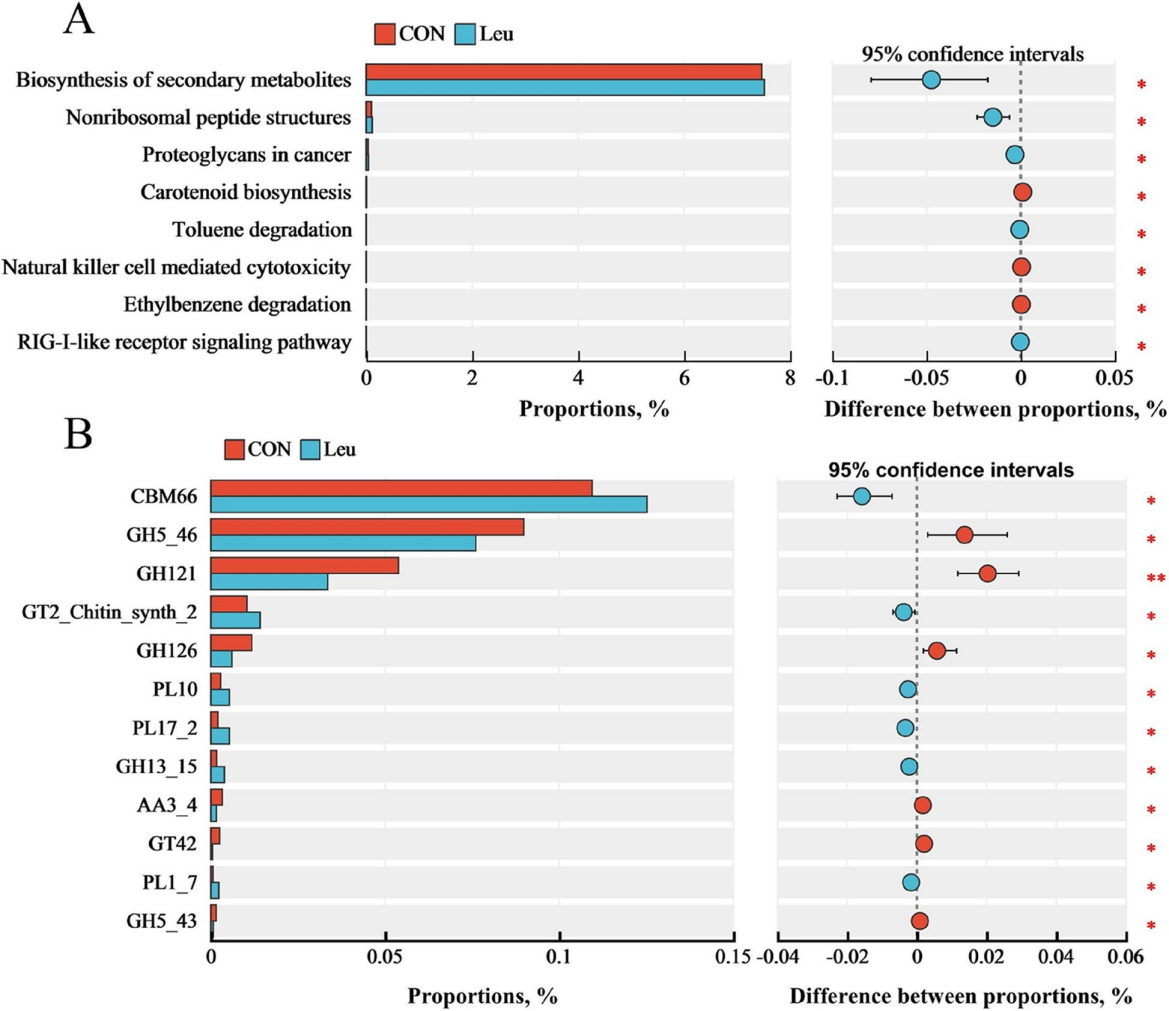
Results of metagenomic sequencing of the rumen bacteria function in the CON and Leu (*n* = 6). **A** Prediction of microbial functional differences based on KEGG database. **B** Comparisons of the abundance of CAZymes genes of rumen microbiomes by the Welch’s *t*-test in metagenomic sequencing. The differences are represented by the color of the group, “CON” means basal diet group; “Leu” means basal diet supplementation with 6.0 g L-Leu/100 kg BW/d group. ^*^P < 0.05, ^**^P < 0.01

Through metagenomic sequencing, 71.92% of the unique genes derived from the rumen microflora were classified into KEGG pathways, and 11.96% into CAZymes. Based on the KEGG database for functional annotation of metagenomic data, Welch’s *t*-test (*P* < 0.05) revealed significant differences in two pathways: biosynthesis of secondary metabolites and nonribosomal peptide structures between the two groups (Fig. 4A). The function of CAZymes helps explore the contribution of microorganisms to carbohydrate metabolism. We found the highest percentages in four major classes: carbohydrate-binding modules (CMBs), polysaccharide lyases (PLs), glycoside hydrolases (GHs), and glycosyltransferases (GTs) at level A (Fig. 4B). At level B, based on Welch’s *t*-test, the following differential enzyme families were found to be higher in the Leu group (*P* < 0.05): CBM66, GT2-Chitin-synth-2, PL10, PL17-2, GH13-15, and GT63. Meanwhile, GH5-45, CBM40, GH121, GH126, GT42, and GH5-43 were found to be higher in the CON group (*P* < 0.05).

### Metabolites analysis of ruminal fluid

**Fig. 5:**
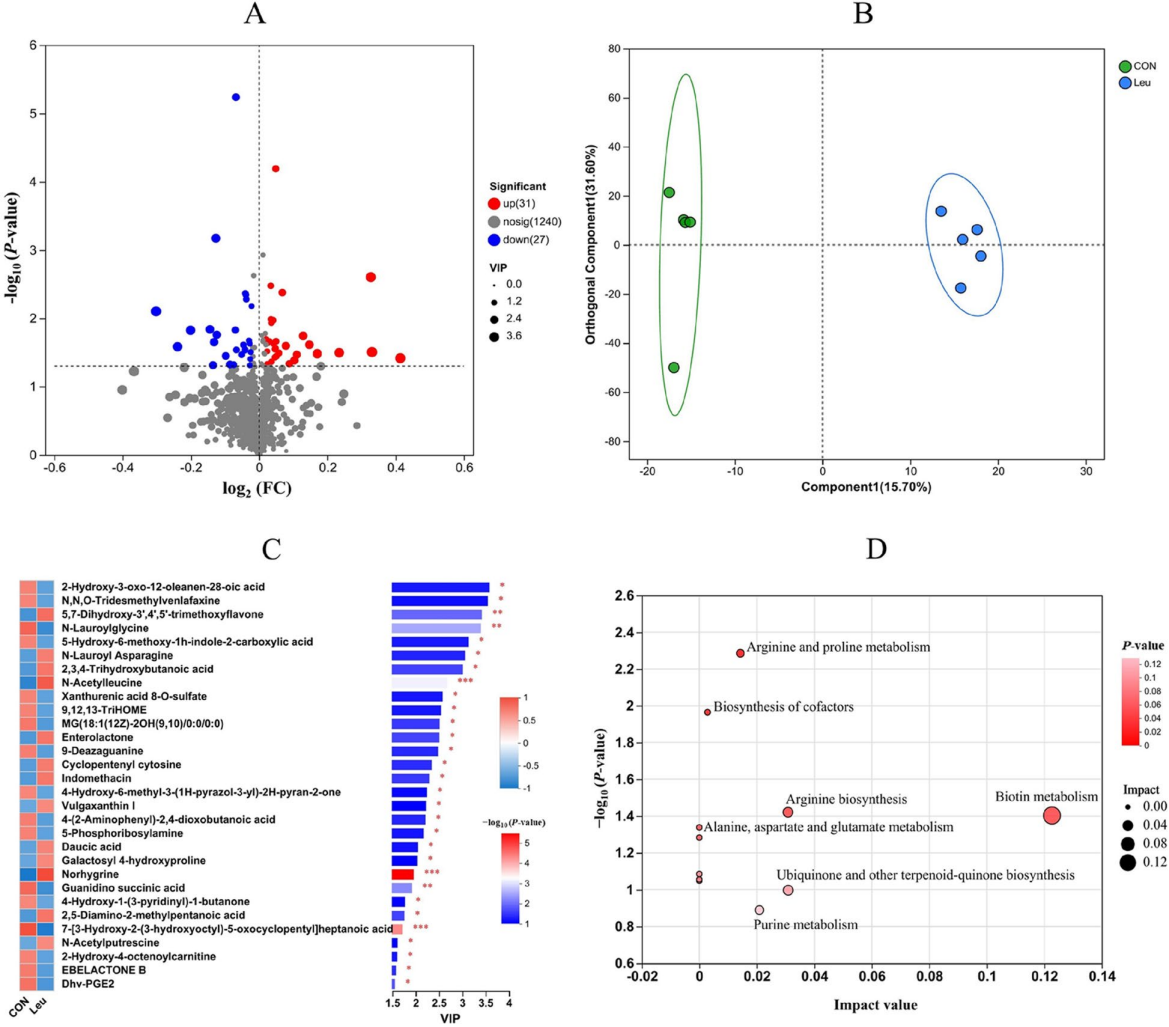
Results of rumen metabolites analysis (*n* = 5). **A** The ruminal differential metabolites as affected by Leu. **B** Principal component analysis (OPLS-DA) of rumen metabolites. **C** The Top 30 rumen differential metabolites by KEGG compound database (CON/Leu, variable importance in the projection [VIP] > 1.5, *P* < 0.05). **D** Metabolic pathways were analyzed based on different metabolites (Impact > 0.2, *P* < 0.05). The differences are represented by the color of the group, “CON” means basal diet group; “Leu” means basal diet supplementation with 6.0 g L-Leu/100 kg BW/d group

After rigorous quality screening and identification, we obtained 58 reliable metabolites across all samples (Fig. 5A). Combined with statistical analysis and the VIP values obtained from the OPLS-DA analysis (Fig. 5B and C), the top 30 of these differential rumen metabolites were further classified according to their properties, which are mainly distributed among organic acids, lipids, and hormones and transmitters (Additional file 2: Fig. S3). According to the pathway topology analysis, five metabolic pathways were significantly enriched by differential metabolites (Fig. 5D, *P* < 0.05), including biotin metabolism, biosynthesis of cofactors, arginine biosynthesis, arginine and proline metabolism, alanine, and aspartate and glutamate metabolism.

### Correlations between rumen fermentation parameters, rumen bacterial biomarkers, and rumen epithelium gene expression

**Fig. 6:**
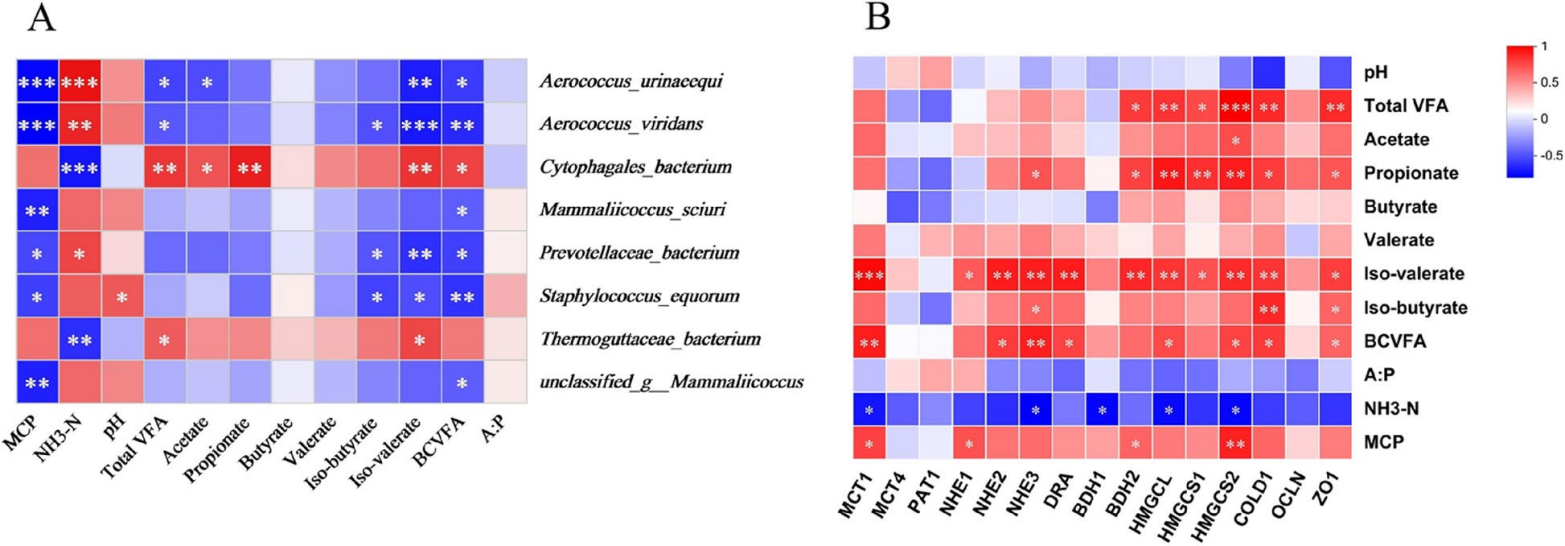
The Spearman correlations between the rumen fermentation parameters, rumen bacterial biomarkers, and rumen epithelium gene expression. **A** Spearman correlation analysis of differential species and rumen fermentation parameters. **B** Spearman correlation analysis of rumen fermentation parameters and genes involved in VFA absorption and metabolism of rumen papilla. ^*^P < 0.05, ^**^P < 0.01, ^***^P < 0.001. *VFA* Volatile fatty acid, *BCVFA* Branched-chain VFA, *A:P* Acetate to propionate ratio, *NH*_*3*_*-N* Ammonia nitrogen, *MCP* Microbial crude protein, *MCT1/4* Monocarboxylate transporter isoform 1/4, *PAT1* Putative anion transporter isoform 1, *DRA* Downregulated in adenoma, *NHE1/2/3* Sodium/proton exchanger isoform 1/2/3, *BDH 1/2* Beta-hydroxybutyrate dehydrogenase 1/2, *HMGCL* 3-Hydroxy-3-methylglutaryl-CoA lyase, *HMGCS 1/2* 3-Hydroxy-3-methylglutaryl-CoA synthase isoform 1/2, *CLDN1* Claudin 1, *OCLN* Occludin, *ZO1* Zonula occluden 1

To explore potential microbial functions, Spearman correlations were constructed between the microorganism biomarkers and rumen fermentation parameters (Fig. 6A). The MCP concentration was negatively correlated with *Aerococcus_urinaeequi*, *Aerococcus_viridans*, *Mammaliicoccus_sciuri*, *Prevotellaceae_bacterium*, *Staphylococcus_equorum*, and *unclassified_g_Mammaliicoccus* (*P* < 0.05). The NH_3_-N concentration was positively correlated with *Aerococcus_urinaeequi*, *Aerococcus_viridans*, and *Prevotellaceae_bacterium* (*P* < 0.05), while negatively correlated with *Cytophagales_bacterium* and *Thermoguttaceae_bacteriu*m (*P* < 0.05). The concentrations of isovalerate and BCVFA were both negatively correlated with *Aerococcus_urinaeequi*, *Aerococcus_viridans*, *Prevotellaceae_bacterium*, and *Staphylococcus_equorum* (*P* < 0.05).

The Spearman correlation analysis shows that the concentrations of total VFA, propionate, and isovalerate are positively correlated with the relative abundance of *ZO1*, *COLD1*, *HMGCS1*, *HMGCS2*, *HMGCL*, and *BDH2* (*P* < 0.01) (Fig. 6B). Additionally, the concentrations of isovalerate and BCVFA are positively correlated with the relative abundance of *DRA*, *NHE3*, *NHE2*, and *MCT1* (*P* < 0.01). A negative correlation was also found between the NH_3_-N concentration and the relative abundance of *HMGCS2*, *HMGCL*, *BDH1*, *NHE1*, and *MCT1* (*P* < 0.05). However, the MCP concentration is positively correlated with the relative abundance of *HMGCS2*, *BDH2*, *NHE1*, and *MCT1* (*P* < 0.05).

## Discussion

### Rumen fermentation parameters

Dietary crude protein or amino acids can be extensively degraded into peptides, amino acids, CO_2_, and NH_3_ by ruminal microorganisms. Like other essential amino acids, L-Leu is degraded during rumen fermentation [7]. However, what it is degraded into and its effect on rumen function in beef cattle is rarely reported. The results of the present experiment showed that supplementing with L-Leu increased the concentrations of total VFA, propionate, iso-valerate, and BCVFA at 4 h post-feeding, but did not affect the dynamics of rumen fluid pH, similar to findings by others [9, 35, 36]. This suggests that supplementing with L-Leu potentially balances ruminal pH by regulating the uptake and metabolism of VFAs in the rumen epithelium. Stable and normal rumen pH ensures rumen microbial activity and fermentation efficiency. Available studies have confirmed that leucine and iso-valerate are directionally converted into each other in the rumen [5, 37]. Additionally, iso-valerate promotes the growth of fiber-degrading bacteria and MCP synthesis [7, 11], and may potentially promote the development of the rumen epithelium. The results of the present experiment also showed that supplementing with L-Leu increased the MCP concentration at 4 h post-feeding, while decreasing ruminal NH_3_-N, which is consistent with our previous in vitro rumen fermentation results [7]. This suggesting that the utilization of ruminal NH_3_-N is enhanced and potentially converted to MCP. Ruminal MCP accounts for 50% to 80% of total absorbable protein, and Leu is an important component of MCP [38]. Metabolizable microbial crude protein moves into the lower digestive tract with digesta and absorbed into bloodstream, which may be an effective pathway for increasing the free Leu level in serum.

Additionally, the carbohydrate-active enzyme (CAZyme) database analysis showed that supplementing with L-Leu increased the relative abundance of CBM66, GH13_15, GT2_Chitin_synth_2, GT63, PL10, and PL17_2 in the present experiment. Available studies have confirmed that polysaccharide lyases (PL) catalyze the breaking of glycosidic bonds within the polysaccharide molecules through the *β*-elimination mechanism to produce oligosaccharides, and the carbohydrate-binding module (CBM) plays an important role in enhancing the catalytic activity of additional CAZymes [39]. The GH13 family, also known as the *α*-amylase family, is the largest family of glycoside hydrolases in CAZymes [40]. This suggests that Leu may promote the degradation of non-structural carbohydrate materials by regulating CAZyme abundance in the rumen. This may partly explain how Leu promotes ruminal propionate production.

### Rumen microorganism composition and metabolites

The changes in rumen fermentation parameters and fermentation patterns were closely related to the structure of rumen microbes and their metabolites [41]. The results of the present experiment showed that supplementing with L-Leu did not affect the richness and diversity (Chao1 and Shannon index) of the rumen bacterial community in beef cattle. At the phylum level, the dominant ruminal bacteria were found to be Bacteroidota and Bacillota, bacteria associated with degradation of non-fiber materials (crude protein) [42]. However, the effect was not significant, which is inconsistent with the results of our previous in vitro rumen fermentation trial. This may be related to the physiological condition of beef cattle and feed formulation. Additionally, the relative abundance of Planctomycetota, Candidatus Melainabacteria, and Thermotogota at the phylum level was increased by Leu. Combined with the results of the LEfSe, it was found that *Mammaliicoccus_sciuri*, *Aerococcus_urinaeequi*, *Staphylococcus_equorum*, and *Aerococcus_viridans* belong to the Bacillota phylum, while *Thermoguttaceae_bacterium* belongs to the Planctomycetota phylum. Available studies have indicated that the Planctomycetota phylum can effectively degrade fiber feeds [43, 44], and the Bacillota phylum can reduce the growth of pathogenic bacteria.

The results of the present experiment showed that supplementing with L-Leu upregulated and downregulated 31 and 27 differential rumen metabolites, respectively. Additionally, the top 30 of these differential metabolites were mainly distributed among organic acids, lipids, and hormones and transmitters. This suggests that Leu can alter ruminal metabolites through the amino acid metabolism pathway and the cofactors and vitamins metabolism pathway. The results of the present experiment also showed that supplementing with L-Leu increased the concentration of essential amino acids (phenylalanine, lysine, threonine, isoleucine) and nonessential amino acids (cystine, tyrosine, histidine) in the rumen. These changes were similar to the results of our previous in vitro bovine rumen fermentation study [7]. Moreover, ruminal amino acids and VFAs, as metabolites of feed degradation by rumen microorganisms, can in turn affect rumen microorganism growth [45, 46]. Therefore, leucine treatment, rumen microorganisms, and rumen metabolites form a virtuous cycle that improves rumen fermentation.

### Rumen epithelial development

Although Leu treatment increased the ruminal concentrations of total VFA and individual VFAs in beef cattle, the rumen fluid pH remained within the normal range. This may be related to Leu’s regulation of the rate at which rumen epithelial cells uptake and metabolize volatile fatty acids, though further validation is required [23, 34]. Considering that the concentrate-to-roughage ratio of the experimental diet was 7:3, the integrity of the rumen epithelial papillae is extremely important for the absorption of VFA. The results of the present experiment showed that supplementing with L-Leu increased the mRNA abundance of *CLDN1*, *OCLN*, and *ZO1*, which are involved in barrier function and tight junctions. VFA absorption and metabolism are one of the most important physiological functions of the ruminal epithelium, playing a pivotal role in the energy supply of ruminants [6, 21]. The results of the present experiment also showed that supplementing with L-Leu increased the mRNA abundance of *MCT1*, *NHE2*, *NHE3*, *DRA*, *BDH2*, *HMGCL*, *HMGCS1*, and *HMGCS2*. Available studies have indicated that *MCT1* and *DRA* can improve VFA absorption, *NHE2* and *NHE3* can regulate the intracellular pH of rumen papillae, and *BDH2*, *HMGCL*, *HMGCS1*, and *HMGCS2* are associated with cholesterol synthesis and ketogenesis in the ruminal epithelium [23, 32, 34]. Ketogenesis effect and cholesterol biosynthesis are the primary pathways of VFA metabolism in rumen epithelium cells [47], providing energy for ruminants. Moreover, Spearman correlation analysis shows that the concentrations of VFAs, particularly isovalerate and BCVFA, were positively correlated with the relative mRNA abundance of genes involved in VFA absorption and metabolism in rumen papillae. This suggests that Leu may improve the barrier function and enhance VFA absorption and metabolism by regulating the relative abundance of key genes in the beef cattle rumen epithelium.

In rumen epithelium papillae, the stratum corneum and stratum granulosum cells are responsible for the absorption and transportation of nutrients, while the stratum spinosum and stratum basal cells have the function of ruminal VFA metabolism and renewal and repair papillae cell [34]. The results of the present experiment also showed that supplementing with L-Leu increased the thickness of the stratum spinosum and stratum basal, while decreasing the thickness of the stratum granulosum. This suggests that ruminal VFA may be transported to the stratum spinosum more quickly, improve VFA metabolism and potentially providing more energy for beef cattle [32, 47]. Previous study reported that dietary supplementation with BCVFAs (iso-butyrate, iso-valerate) can stimulate rumen development in young ruminants [48, 49]. Therefore, we suggest that iso-valerate, as a degradation product of leucine, plays a role in promoting rumen development in mature ruminants. Additionally, the thickness of stratum corneum was not significantly affected by Leu in the present study. Based on the results of changes in the morphologic structure of the rumen epithelium, we conclude that dietary supplementation with Leu can improve the rumen epithelium morphological and promote uptake and metabolism of VFA in the rumen epithelium of fattening beef cattle. Meanwhile, these favorable results may also, to some extent, explain the mechanism by which Leu increased the average daily weight gain of beef cattle in our previous study [16].

## Conclusion

Overall, dietary supplementation with L-Leu improved the rumen fermentation parameters, promoted propionate production, increased rumen MCP content, and enhanced rumen epithelial function in beef cattle. Supplementing with L-Leu enhanced the relative mRNA abundance of genes involved in barrier function and VFA absorption and metabolism in rumen papillae by enhancing the production of total VFA, propionate, isovalerate, and BCVFAs. This study provides novel insights into the effects of L-Leu on rumen fermentation function and rumen epithelium development in fattening beef cattle, as well as a valuable reference for the application of L-Leu.

## Supplementary Information

Additional file 1: Table S1. Primer sequences, genes targeted, and length of PCR products. Table S2. The effects of dietary supplementation L-leucine on messenger RNA (mRNA) expression of genes involved in rumen epithelial absorption, metabolism, and integrity in beef cattle (*n *= 6). Table S3. The quality of metagenome (*n *= 6).

Additional file 2: Fig. S1. The *α*-diversity indices including observed genus, A and B Chao and Shannon indexes (*n *= 6); C Principal coordinates analysis (PCoA). D Non-metric multidimensional scaling (NMDS). Fig. S2. Results of metagenomic sequencing of the rumen bacteria in the CON and Leu (*n *= 6). A Differences in bacterial phylum levels. B Differences in bacterial species levels.^*^P < 0.05, ^**^P< 0.01, ^***^P < 0.001. Fig. S3. Classification of rumen differential metabolites by KEGG compound database (CON/Leu, variable importance in the projection [VIP] > 1.5, *P* < 0.05) (*n *= 5).

## Data Availability

The datasets used and/or analyzed during the current study are available from the corresponding author on reasonable request.

## Abbreviations

AA: Amino acid
ADF: Acid detergent fiber
A:P: Acetate to propionate ratio
BCAA: Branched-chain amino acid
BDH-1/2: Beta-hydroxybutyrate dehydrogenase, isoform 1/2
BW: Body weight
BCVFA: Branched-chain volatile fatty acid
CAZys: Carbohydrate-active enzymes
CP: Crude protein
Ca: Calcium
CLDN1: Claudin 1
DM: Dry matter
DRA: Downregulated in adenoma
EAA: Essential amino acid
EE: Ether extract
FAA: Free amino acid
GAPDH: Glyceraldehyde-3-phosphate dehydrogenase
HMGCL: 3-Hydroxy-3-methylglutaryl-CoA lyase
HMGCS1/2: 3-Hydroxy-3-methylglutaryl-CoA synthase isoform 1 and 2
KEGG: Kyoto encyclopedia of genes and genomes
LEfSe: Linear discriminant analysis effect size
Leu: Leucine
MCP: Microbial crude protein
MCT1/4: Monocarboxylate transporter isoform 1/4
NDF: Neutral detergent fiber
NH_3_-N: Ammonia-N
NHE1/2/3: Sodium/proton exchanger isoform 1/2/3
OCLN: Occludin
PAT1: Putative anion transporter isoform 1
P: Phosphorus
qRT-PCR: Quantitative real-time PCR
TMR: Total mixed ration
VFA: Volatile fatty acid
ZO1: Zonula occluden 1
