# Supplementary material for: Dietary L-leucine supplementation improves ruminal fermentation parameters and epithelium development in fattening Angus beef cattle
Source: J Anim Sci Biotechnol. 2025 Apr 23;16:60. doi: 10.1186/s40104-025-01190-0 (PMC12020287; doi:10.1186/s40104-025-01190-0)
Supplement: Supplementary file 1 — Additional file 1: Table S1. Primer sequences, genes targeted, and length of PCR products. Table S2. The effects of dietary supplementation L-leucine on messenger RNA (mRNA) expression of genes involved in rumen epithelial absorption, metabolism, and integrity in beef cattle (n = 6). Table S3. The quality of metagenome (n = 6). [file 40104_2025_1190_MOESM1_ESM.docx]

**Additional file 1**

**Table S1** Primer sequences, genes targeted, and length of PCR products

| **Gene name** | **Gene ID** | **Primer sequences1 (5′→3′)** | **Amplicon Size (bp)** |
| --- | --- | --- | --- |
| *MCT1* | NM_001037319.1 | F: AGCCGCGTATAACGATACTTG  R:TCCAATTACCACTGCCCACC | 133 |
| *MCT4* | NM_001109980.3 | F:GAGCGGCAGCAGGGAG  R:TGACAGAGGCTGTTCCAAGG | 148 |
| *PAT1* | NM_001076852.2 | F:CTCTTGCCCGAGCTTGGTAG  R:TGTCCCTCTCTCCCGGC | 81 |
| *NHE1* | NM_174833.2 | F:GTCAGGCATCATGGCACTCA  R:TGATGGTCGTGTGGGACTTG | 89 |
| *NHE2* | XM_002691185.7 | F:ATCCGTCAGCGAACCTTGTC  R:AAGCTGCTGTCCTTCCGAAT | 128 |
| *NHE3* | NM_001192154.2 | F:GGGATCGAGTTCCTGGCAAA  R:GCAGGGTTGTCAATTCCTGAG | 71 |
| *DRA* | NM_001083676.1 | F:AAAGTTGTGCCGCTGTTCTG  R:GCGATATGCTGGCAACCAAG | 85 |
| *HMGCL* | NM_001075132.1 | F:GACCCTCCGGGCTGTCA  R:CGTCTCGAGGACCAACTTCC | 80 |
| *HMGCS1* | NM_001206578.1 | F:GGTCCGCGGCTATAAAGCTA  R:TCTCCTCCTTCAGGCACCAA | 113 |
| *HMGCS2* | NM_001045883.1 | F:TGACTTCCTGTTGGCCAGTG  R:AGCTTTAGTCCCCTGAAGGC | 75 |
| *BDH1* | NM_001034600.2 | F:CTGCGAAGCGCCAGGTTATC  R:CTCCTGGTGGGTTCCCAAAAC | 90 |
| *BDH2* | NM_001034488.2 | F:TTGCTGCTCTAAAATGTGACG  R:TATGCCTCTGACCACACAGC | 71 |
| *CLDN1* | NM_001001854.2 | F: TGCTGAATCTGAACAGCACT  R: CTCGTCGTCTTCCATGCACT | 128 |
| *OCLN* | NM_001082433.2 | F: ATACCACTCCTCCTCCCTAGC  R: TGAGATCAGCCAATCTGCG | 129 |
| *ZO1* | XM_024982006.2 | F: GTCCATGACTCCTGACGGTT  R: GGTTTTAGGATCACCCGACGA | 70 |
| *GAPDH* | [NM_001034034.2](https://www.ncbi.nlm.nih.gov/entrez/viewer.fcgi?db=nucleotide&id=402744670) | F: AGGTCGGAGTGAACGGATTC  R: ATGGCGACGATGTCCACTTT | 85 |

The PCR amplification effciencies for all of the primers ranged between 96 and 100%

**Table S2** The effects of dietary supplementation L-leucine on messenger RNA (mRNA) expression of genes involved in rumen epithelial absorption, metabolism, and integrity in beef cattle (*n*=6)

| **Item** | **Treatment** | | **SEM** | ***P*-value** |
| --- | --- | --- | --- | --- |
|  | **CON** | **Leu** |  |  |
| *MCT1* | 1 | 2.16 | 0.22 | 0.002 |
| *MCT4* | 1 | 1.37 | 0.19 | 0.346 |
| *PAT1* | 1 | 0.91 | 0.13 | 0.737 |
| *NHE1* | 1 | 1.66 | 0.21 | 0.120 |
| *NHE2* | 1 | 2.16 | 0.24 | 0.008 |
| *NHE3* | 1 | 2.14 | 0.23 | 0.004 |
| *DRA* | 1 | 4.70 | 0.90 | 0.032 |
| *BDH1* | 1 | 1.37 | 0.12 | 0.136 |
| *BDH2* | 1 | 2.29 | 0.21 | 0.000 |
| *HMGCL* | 1 | 2.08 | 0.22 | 0.005 |
| *HMGCS1* | 1 | 2.81 | 0.33 | 0.001 |
| *HMGCS2* | 1 | 6.15 | 0.91 | 0.001 |
| *COLD1* | 1 | 3.17 | 0.51 | 0.031 |
| *OCLN* | 1 | 3.67 | 0.68 | 0.042 |
| *ZO-1* | 1 | 1.91 | 0.21 | 0.030 |

**Table S3** The quality of metagenome (*n*=6)

| **Sample** | **Clean reads** | **Optimized reads** | **Contigs** | **N50, bp** | **N90, bp** | **ORF** | **Total length, bp** | **Average length, bp** |
| --- | --- | --- | --- | --- | --- | --- | --- | --- |
| A1 | 43,904,604 | 43,518,652 | 642,128 | 596 | 345 | 802,425 | 345,624,669 | 430.73 |
| A2 | 49,324,798 | 48,910,866 | 739,653 | 587 | 343 | 917,143 | 391,151,253 | 426.49 |
| A3 | 44,340,052 | 43,949,240 | 663,988 | 564 | 341 | 807,664 | 337,611,843 | 418.01 |
| A4 | 45,437,350 | 45,049,604 | 731,522 | 603 | 346 | 906,077 | 397,721,397 | 438.95 |
| A5 | 46,220,584 | 45,839,754 | 683,527 | 564 | 341 | 836,685 | 350,193,810 | 418.55 |
| A6 | 46,631,310 | 46,239,588 | 712,795 | 586 | 343 | 898,315 | 377,962,800 | 420.75 |
| B1 | 46,932,906 | 46,528,730 | 717,621 | 565 | 341 | 871,660 | 366,114,180 | 420.02 |
| B2 | 46,800,670 | 46,400,642 | 735,970 | 604 | 346 | 916,347 | 399,280,728 | 435.73 |
| B3 | 43,744,672 | 43,378,218 | 578,621 | 596 | 344 | 727,380 | 312,338,094 | 429.4 |
| B4 | 45,888,368 | 45,478,028 | 736,802 | 543 | 339 | 896,307 | 370,840,794 | 413.74 |
| B5 | 45,639,676 | 45,244,798 | 677,460 | 617 | 348 | 854,927 | 377,644,305 | 441.73 |
| B6 | 45,177,238 | 44,774,336 | 654,254 | 576 | 343 | 802,545 | 340,915,179 | 424.79 |

*ORF* Open reading fram
