# Supplementary material for: Dietary L-leucine supplementation improves ruminal fermentation parameters and epithelium development in fattening Angus beef cattle
Source: J Anim Sci Biotechnol. 2025 Apr 23;16:60. doi: 10.1186/s40104-025-01190-0 (PMC12020287; doi:10.1186/s40104-025-01190-0)
Supplement: Supplementary file 2 — Additional file 2: Fig. S1. The α-diversity indices including observed genus, A and B Chao and Shannon indexes (n = 6); C Principal coordinates analysis (PCoA). D Non-metric multidimensional scaling (NMDS). Fig. S2. Results of metagenomic sequencing of the rumen bacteria in the CON and Leu (n = 6). A Differences in bacterial phylum levels. B Differences in bacterial species levels.*P < 0.05, **P< 0.01, ***P < 0.001. Fig. S3. Classification of rumen differential metabolites by KEGG compound database (CON/Leu, variable importance in the projection [VIP] > 1.5, P < 0.05) (n = 5). [file 40104_2025_1190_MOESM2_ESM.docx]

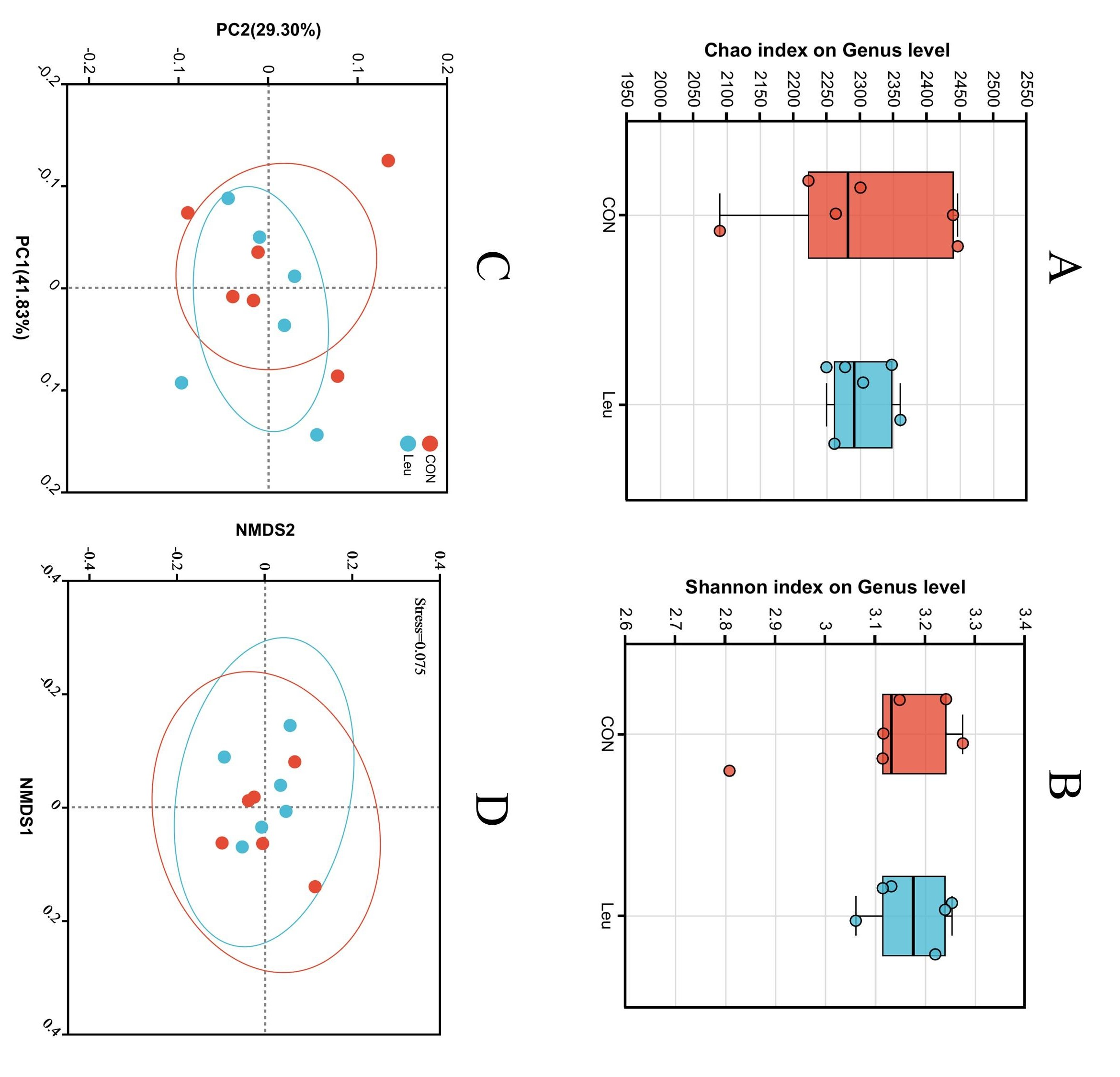


**Fig. S1** The *α*-diversity indices including observed genus, **A** Chao and **B** Shannon indexes (*n*=6); **C** Principal coordinates analysis (PCoA); **D** non-metric multidimensional scaling (NMDS)


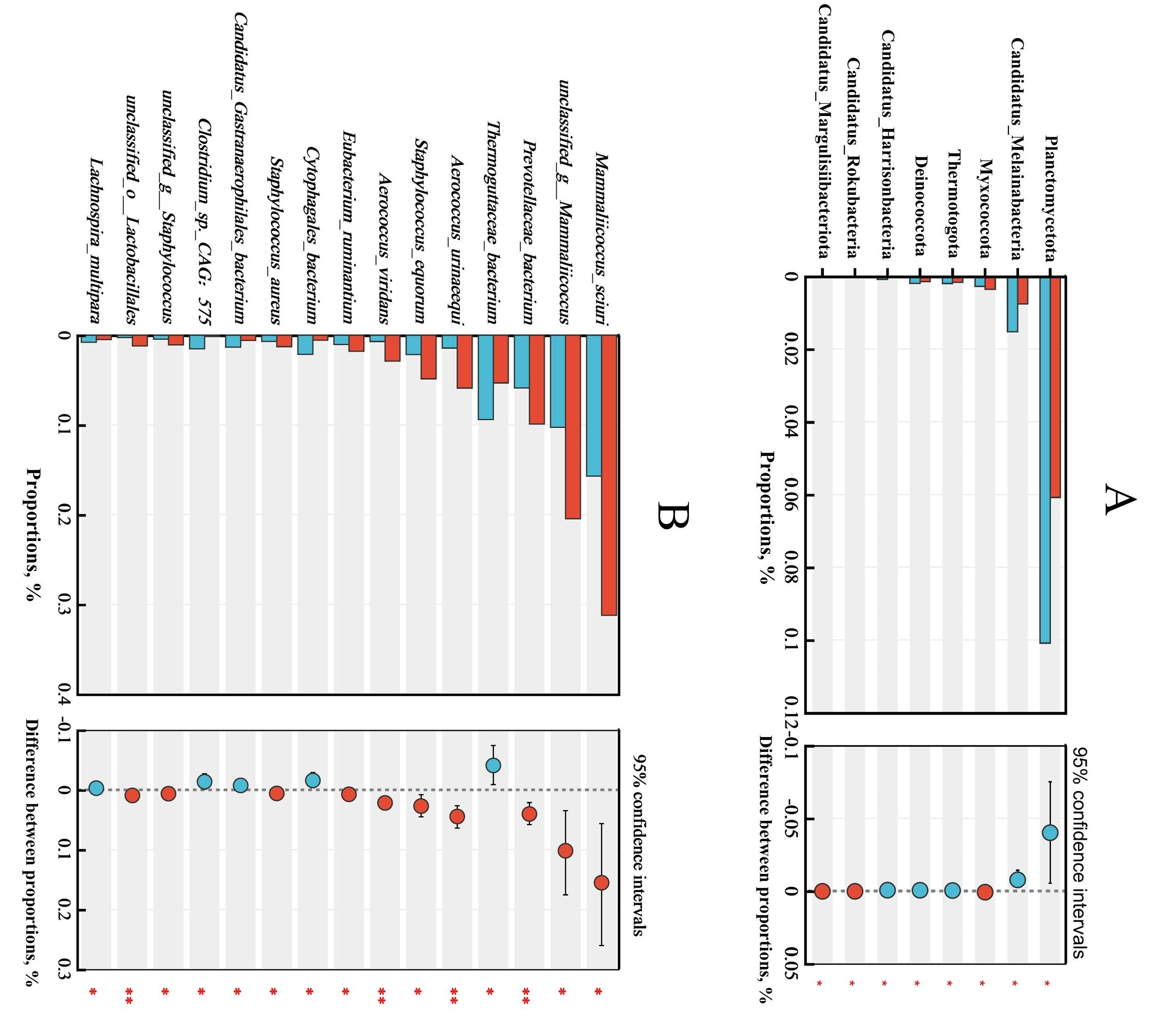


**Fig. S2** Results of metagenomic sequencing of the rumen bacteria in the CON and Leu (*n*=6). **A** Differences in bacterial phylum levels; **B** Differences in bacterial species levels. ^*^*P* < 0.05, ^**^*P* < 0.01, ^***^*P* < 0.001


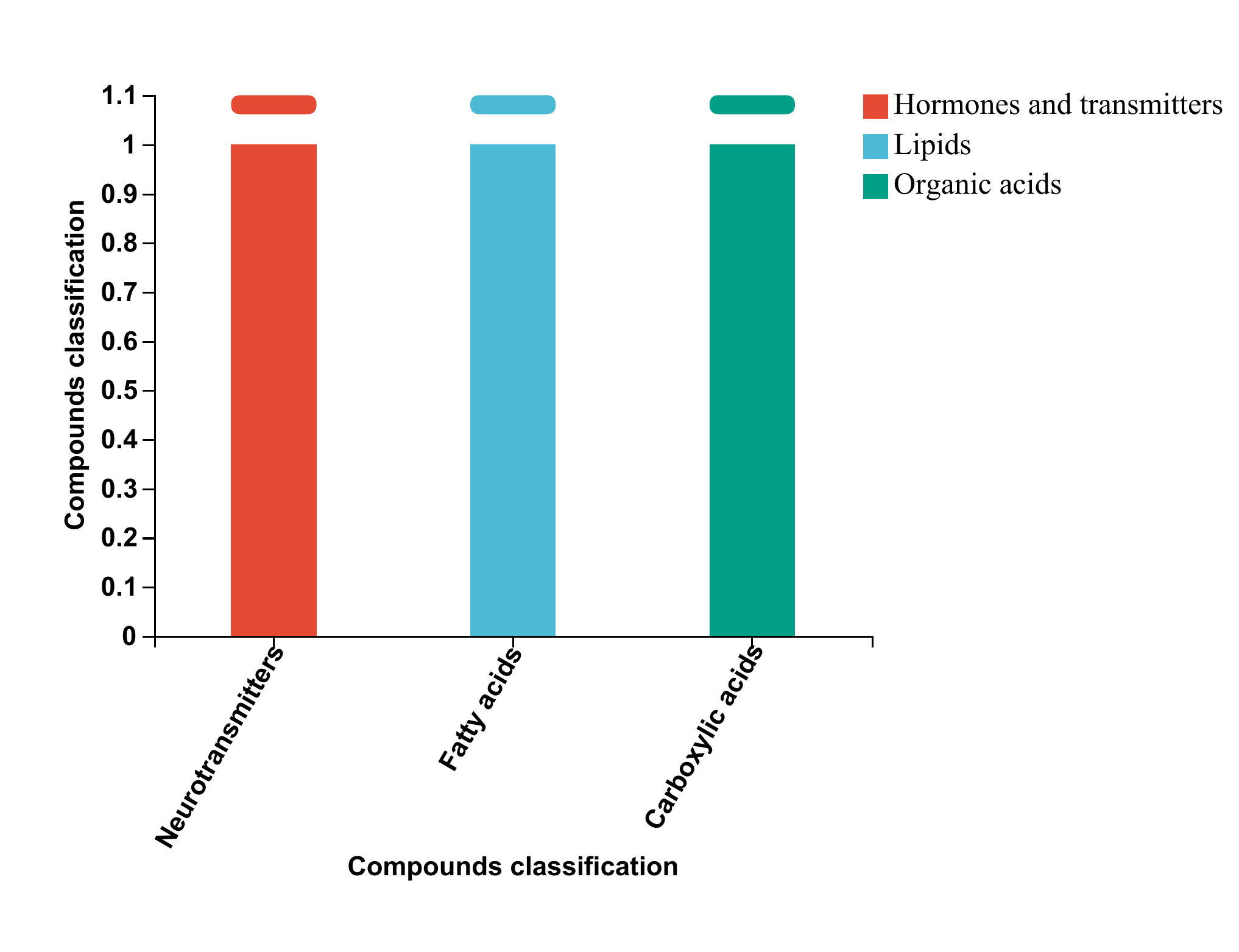


**Fig. S3** Classification of rumen differential metabolites by KEGG compound database (CON/Leu, variable importance in the projection [VIP] > 1.0, *P* < 0.05) (*n*=5)
